# Rhinovirus—A True Respiratory Threat or a Common Inconvenience of Childhood?

**DOI:** 10.3390/v15040825

**Published:** 2023-03-24

**Authors:** Sunčanica Ljubin-Sternak, Tomislav Meštrović

**Affiliations:** 1 Clinical Microbiology Department, Teaching Institute of Public Health, “Dr Andrija Štampar”, 10000 Zagreb, Croatia; sljsternak@stampar.hr; 2 Medical Microbiology Department, School of Medicine, University of Zagreb, 10000 Zagreb, Croatia; 3 University Centre Varaždin, University North, 42000 Varaždin, Croatia; 4 Institute for Health Metrics and Evaluation and the Department of Health Metrics Sciences, University of Washington, Seattle, WA 98195, USA

**Keywords:** rhinovirus, respiratory pathology, common cold, community-acquired pneumonia, bronchiolitis, wheezing, asthma, virology

## Abstract

A decade-long neglect of rhinovirus as an important agent of disease in humans was primarily due to the fact that they were seen as less virulent and capable of causing only mild respiratory infections such as common cold. However, with an advent of molecular diagnostic methods, an increasing number of reports placed them among the pathogens found in the lower respiratory tract and recognized them as important risk factors for asthma-related pathology in childhood. As the spread of rhinovirus was not severely affected by the implementation of social distancing and other measures during the coronavirus disease 2019 (COVID-19) pandemic, its putative pathogenic role has become even more evident in recent years. By concentrating on children as the most vulnerable group, in this narrative review we first present classification and main traits of rhinovirus, followed by epidemiology and clinical presentation, risk factors for severe forms of the disease, long-term complications and the pathogenesis of asthma, as well as a snapshot of treatment trials and studies. Recent evidence suggests that the rhinovirus is a significant contributor to respiratory illness in both high-risk and low-risk populations of children.

## 1. Introduction

Rhinovirus (RV) is widely recognized as the primary cause of a common cold, a clinical condition characterized by a combination of symptoms such as nasal congestion, rhinorrhea, cough, sore throat and general discomfort [1]. Albeit a relatively common and mild, self-limiting syndrome, its potential as a causative agent of severe illness has often been overlooked [2,3,4]. Recent research endeavors have focused on the impact of RV infection on the development of lower respiratory tract infections (LRTIs) and respiratory complications in children, such as wheezing and asthma. In the following sections, we will discuss what the latest literature has to say on RVs biological features, its pathogenesis, modes of transmission, the activation of host immune response, clinical presentation and risk factors that predispose affected individuals to develop severe forms of infection, potential long-term consequences of the infection, as well as a snapshot of treatment trials and studies in this field. In other words, our primary aim is to explore the true range of respiratory morbidity in children infected with RV.

## 2. Classification and Main Characteristics

Rhinoviruses (RVs) were first discovered in 1956 through isolation on monkey kidney cells by two independent research groups [5,6]. In 2006, a new group of RVs was discovered using molecular techniques [7]. Initially, they were classified into around 100 consecutive serotypes, but are now genetically categorized into three species, Rhinovirus A (RV-A), Rhinovirus B (RV-B) and Rhinovirus C (RV-C), within the *Enterovirus* genus of the *Picornaviridae* family [3,7]. The serotype classification based on antigenic and cross-neutralization properties has been replaced with genotype classification based on nucleotide sequence divergence in the viral protein (VP) 1 gene, where different rhinovirus serotypes consistently exhibit more than 11% nucleotide sequence divergence from each other [8]. As of now, nearly 170 genotypes have been recognized, with RV-A comprising 80 types, RV-B comprising 32 types and RV-C comprising 57 types [8]. Rhinovirus species vary in their use of cell entry receptors. More specifically, a majority of RV-A attach to the intercellular adhesion molecule (ICAM)-1 (classified as the major receptor group) while 12 others bind low density lipoprotein receptor (LDL-R) (minor receptor group). RV-B attaches to ICAM-1 and RV-C uses human cadherin-related family member 3 (CDHR3) as its receptor [9,10].

**Figure 1:**
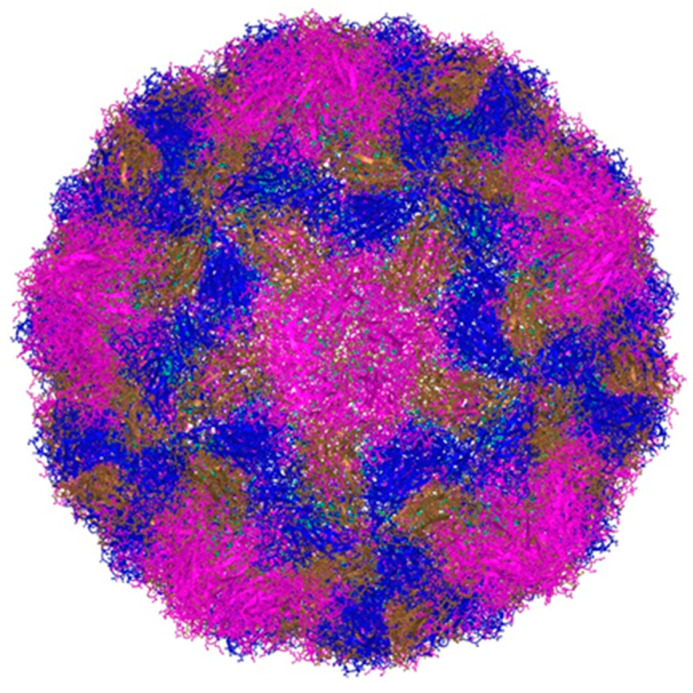
A three-dimensional structure of rhinovirus B14 drug-resistant mutant (original representation; MMDB ID: 225640). Color legend: capsid subunit VP1 (pink), capsid subunit VP2 (brown), capsid subunit VP3 (blue), capsid subunit VP4 (green). Source: National Center for Biotechnology Information (NCBI) Structure Summary MMDB. Representations were made with the use of the iCn3D Structure Viewer using Three.js and jQuery (source code available at https://github.com/ncbi/icn3d, accessed on 21 March 2023).

Rhinoviruses (RVs) are small viruses without an envelope. Their genome is made up of a single-stranded, positive-sense RNA with a single open reading frame (ORF) joined to a 50 untranslated region (50UTR) and a short viral priming protein (VPg) [7]. The ORF encodes a poly-protein, which is cleaved by virally encoded proteases into 11 proteins. The viral capsid is made up of four proteins (VP1, VP2, VP3, and VP4) (Figure 1), responsible for its antigenic diversity, whereas the non-structural proteins are involved in viral genome replication/assembly. RV detection from clinical samples is typically done using a relatively conserved 50 UTR region containing an internal ribosomal entry site (IRES), while more accurate genotyping relies on VP4/VP2 or VP1 sequence analysis [7].

According to the current classification, RV sequence belongs to A, B or C species if it has at least 70% similarity with other subtypes within this species. More than 90% nucleotide similarity between two RV sequences on VP2/VP4 region and/or more than 87% similarity on VP1 region indicate the same subtype. A clinical RV isolate showing a lower sequence identity may present a new subtype, but it should be confirmed by ICTV subcommittee after full sequencing of the VP1 region [11,12]. It should be noted that the more sequence that is compared, the more accurate is the putative classification, so it would be best to apply whole viral genome sequencing wherever possible.

It is well known that RVs replicate best at 33–35 °C in cell culture, thus it was thought that the higher temperature of the lower respiratory tract will hamper RV replication, making them in turn less virulent and unable to cause severe disease. Direct measurements in the lower airways have shown that a temperature of 33–35 °C in large and medium-sized airways represents an ideal temperature for RV replication. On the other hand, small airway temperatures approach a core temperature of 37 °C. Still, it was shown that in vitro lower airway epithelial cells support RV replication much better than cells derived from upper airways [6,13].

## 3. Modes of Transmission and Prevalence of Infection

The transmission of RVs typically takes place via direct exposure and inhalation of respiratory droplets or micro-droplets, although it can also occur through fomites (such as contaminated surfaces and other inanimate objects) or through direct person-to-person contact [2,14]. This is because RV has moderate resistance to common disinfectants such as alcohol hand rubs [15]. Previously, it was believed that RVs did not contribute to lower respiratory tract diseases due to the idea that they replicate more easily at a lower temperature of 33 °C, which is the temperature of the nasal passage, in comparison to the higher temperature of 37 °C [16]. However, it is important to note that the optimal temperature for replication may differ substantially among recognized RV serotypes, and viral replication at 37°C may still result in titers that are high enough to instigate the infection [17,18].

Indeed, this has been confirmed in both clinical and experimental conditions [14,18]. After being intentionally infected in different experiments, RV has been observed in the lower airways through various techniques, including polymerase chain reaction (PCR), immunostaining and in situ hybridization for detecting positive strand viral ribonucleic acid (RNA) [19,20]. With the increasing use of multiplex diagnostic platforms, we now have a greater understanding of the link between respiratory diseases and RV, and recent epidemiological data suggests that this virus is a rather frequent pathogen (or co-pathogen) in young children presenting with acute respiratory infections [18].

More specifically, there is an agreement in the literature that RVs are the leading causes of acute respiratory infections, and are also the most pervasive group of respiratory viruses spanning across all pediatric age groups and in both outpatient and inpatient settings. Their clinical and socioeconomic burden in the community seems to be sizeable [21] and they represent a substantial fraction of the global burden of upper respiratory tract infections (URTIs), reflected in 17.2 billion incident cases in 2019 [22]. Therefore, active surveillance will be increasingly important in adequately defining the full health care burden of these respiratory viruses, while forecasting the burden of URTIs with the use of novel techniques (such as high-dimensional time series data and forecast combinations) will facilitate healthcare resource planning [23].

There is a question as to why we have seen a resurgence of RV in comparison to other respiratory viruses when coronavirus disease 2019 (COVID-19) social distancing measures were relaxed. One answer may be the already mentioned study nature of non-enveloped viruses such as RVs in comparison to enveloped ones (such as respiratory syncytial virus (RSV), influenza virus and parainfluenza viruses), which was evident in a recent study from Japan [24]. Conversely, it was already observed that period drops in RV circulation coincide with peak activity of influenza viruses [25] in a process known as viral interference [18]; hence, the absence of influenza may have prompted a notable increase in RV. Interactions between RVs and other co-circulating respiratory viruses have been shown to influence viral epidemiology both in individual hosts and across the population [26]. Recent studies have also confirmed how SARS-CoV-2 replication is impaired by primary RV infection, while SARS-CoV-2 does not impact RV replication [27].

Three relevant epidemiological meta-analyses were conducted within the last three years, exploring the prevalence of RVs in children with bronchiolitis and community-acquired pneumonia (CAP), as well as in patients with COVID-19 [28,29]. These articles are hitherto the most stringent attempts to discern the proportion of RVs and other respiratory viruses in the aforementioned conditions, by providing pooled estimates and conducting subgroup analyses after stringent literature search. As a result, these are currently the most relevant publications for placing the frequency of RVs into context and are summarized in Table 1.

Kenmoe et al. assessed the prevalence of RVs in children younger than two years of age with bronchiolitis recorded over two decades in the era before the COVID-19 pandemic [28]. Observational studies that reported molecular detection rates of RVs and other respiratory viruses in children with bronchiolitis have been included. RV was the second most common virus in this population after RSV, with pooled prevalence of 19.29% (95% CI 16.67–22.04%), and there were also 7.1%, (95% CI 4.6–9.9) RSV-RV co-detections [28].

Pratt et al. [29] evaluated the prevalence of RVs and other common respiratory pathogens in children 18 years of age or younger presenting with community-acquired pneumonia (CAP). They estimated an overall pooled prevalence of respiratory virus-associated childhood CAP at 55.0% (95% CI 50.4–59.7), while RVs were found in 22.1% (95% CI 19.5–24·7%) of them (although the complexity of the estimation process was increased by the substantial heterogeneity between studies). In addition, pooled prevalence was higher when using polymerase chain reaction (PCR) assay, whereas the variation in prevalence by the WHO region, national income and under-5 mortality rates were not significant [29].

Krumbein et al. appraised the prevalence of RVs and other community acquired co-infectious agents, as well as relevant risk factors in pediatric and adult patients with COVID-19 [30]. RVs and other enteroviruses accounted for 25.1% of all co-infections (second only to influenza virus), with the pooled prevalence (based on the number of performed tests) of 1.32% (95% CI 1.15–1.52%). Subgroup analyses revealed that co-infection was significantly more frequent in children than adult patients, also with more frequent fatal outcomes [30].

## 4. The Link between Rhinovirus Epidemiology and Clinical Presentation

Over the years, an increasing number of studies and reports have implied that RV may play a pivotal role in symptomatic upper respiratory tract infections (URTIs) in children [31,32,33,34], with its involvement as the principal or at least significant etiologic agent in more than 50% of URTIs [2]. Even more importantly, RVs can have a role in sinusitis and rhinosinusitis, with purported links to resulting chronic conditions. Basharat et al. found that a higher prevalence of RV infections in those with chronic rhinosinusitis, coupled with inflammatory reactions, may prompt exacerbations and progression of this condition [35]. Hence, further studies should also include more insights into host-virome response to obtain better insights into URTIs caused by RVs and its complications.

Different studies showed that RV can be also considered one of the most prominent causes of lower respiratory tract infections (LRTIs), such as pneumonia, bronchiolitis and several forms of severe respiratory disease [36,37], alongside RSV in infants/children and influenza in older individuals [38,39,40]. In a study from Croatia, more than half of all children infected with RV presented with clinically relevant symptoms reflecting LRTI, and in 60.4% of cases RV was detected as a single infection (mono-infection) [37]. A more frequent antibiotic prescription was observed in some instances, particularly in those infected with RV-A species [37].

These findings are reinforced by one European study that was conducted on children under the age of 14 who were diagnosed with community-acquired pneumonia (CAP) following radiological confirmation [41]. The study reported that RV was detected in 29% of children in the studied population, with 40% of these cases also harboring two or more viruses concurrently. Among toddlers, multiple viral infections were found to be implicated as a cause of pneumonia, with RVs playing a significant role; however, clinical severity did not seem to be affected in this age group [2]. In a study from Finland, RV was found to be responsible for 26% of pneumonias, and RV-positive children presented with a higher neutrophil count than those without the virus [42].

Although the focus is mostly on pediatric patients, they are not the only ones affected by RV. Bahabri and colleagues have recently studied 106 hospitalized adult individuals with RV-induced CAP and found that most of them had certain predisposing conditions, such as hypertension, diabetes and chronic respiratory disease [43]. In addition, severe presentation has been observed in those who were hospitalized in the ICU, such as hemoptysis, tachypnea and low number of lymphocytes. Earlier literature sources have already pointed out how RV can be viewed as a significant pathogen in basically all age groups [44].

Moreover, it has been suggested that RV can trigger asthmatic flares in both adults and children, underscoring its potential for causing much greater morbidity than previously recognized [2,45]. RV is recognized as the second most common cause of viral bronchiolitis, following RSV as the most pervasive one, and it can contribute to 68.5% of virus-induced asthma exacerbations in children [45]. This is reflected in different studies that have accentuated the role of RVs in the occurrence of wheezing during the early years of life [46,47,48,49,50]. It is estimated that 20–40% of infants under one year of age who are diagnosed with bronchiolitis are infected or co-infected with RV, and this rate increases to about 50% when hospitalized infants under three years of age are considered [51]. These findings suggest that RV is a significant contributor to respiratory illness in young children and should be taken into account when evaluating URTIs, LRTIs and bronchiolitis.

Additionally, it is well established in the literature that RV-induced wheezing illness can be viewed as a robust predictor of succeeding wheezing and asthma, particularly in two identified high-risk populations: early wheezers presenting with atopic background and pediatric patients hospitalized for early onset wheezing [2,52,53,54]. More specifically, RV infection and blood eosinophils higher than 400 cells per μL are identified as the major risk factors for recurrent wheezing [54]. These findings imply that RV can have a substantial impact on the overall respiratory health of children; hence, the identification and management of RV infections are crucial for the treatment and prevention of respiratory diseases such as pneumonia and asthma.

Studies of high-risk birth cohorts have consistently found RV-induced bronchiolitis and wheezing to be a strong risk factor for school-aged asthmatic illness, further supporting the notion that RV plays a significant role in the development of asthma [55,56]. One multi-center cohort study analyzing 921 infants under one year of age unveiled three different profiles of severe bronchiolitis; those negative for RSV arose mostly due to RV and were related to a history of breathing disorders and/or eczema during infancy, as well as to a higher blood eosinophil count [57].

In addition to its high prevalence in respiratory infections, RV has been shown to cause a lower amount of structural damage to the airways than RSV, but may still contribute to bronchial hyper-reactivity in predisposed patients [2]. Host factors—such as allergy and atopy in family history, environmental exposures (including airway microbiome), immune function, and tobacco smoke or pollution—may also influence the onset and severity of subsequent wheezing and asthma disease [58,59,60].

We also have to be cognizant that finding the viral agent in nasopharynx of a child presenting with CAP does not automatically imply that it is the actual cause. More specifically, it is possible that the virus is simply a coexistent upper airways infection, or the affected individual may be a carrier of the virus without actually being ill. Another possibility is that the child is still shedding the virus from a previous infection, which may not have caused any symptoms. This is particularly relevant in the case of RVs, as studies have shown that 12–22% of people who are not experiencing any symptoms may still have RVs in their respiratory secretions [41,61]. Therefore, it is important to carefully consider all possible explanations for viral presence in a child with CAP before determining whether it is the true cause of their illness.

## 5. Risk Factors for the Development of a Severe Form of the Infection

Several factors linked to the virus, host and environment contribute to the risk of experiencing more severe RV illnesses [62].

There are indications that greater virulence of the virus may be a feature of some rhinovirus species. It has been shown that RV-B types exhibit slower and less efficient replication, as well as lower levels of cellular cytotoxicity and cytokine/chemokine production compared to RV-A or RV-C. This could account for the milder symptoms typically seen with RV-B infections [63]. However, the data on the severity of the clinical picture and the link to the type of rhinovirus are contradictory. Some studies suggest no difference, while others suggest a potential association, particularly with RV-C or even more with certain types of the virus [64]. For instance, Brunning and co-workers showed that in children with respiratory distress who required intensive care unit-admission the distribution of RV species did not differ significantly from the non-hospitalized children [65]. Conversely, more recent studies showed that severe illnesses, low oxygen saturation, cough and wheezing were more common in patients with RV-C than in those with RV-A or RV-B. In addition, when comparing the severity of symptoms presented between RV-C clades, episodes caused by GAC1 and GAC2 clades of RV-C were associated with more severe symptoms than those caused by RV-C (C) and A2 [66].

A genetic tendency towards contracting the rhinovirus (RV) infection has been identified, which can affect the intensity of the disease and the likelihood of rhinovirus-triggered exacerbations. More specifically, variations within a specific location on chromosome 17q21 have been linked to RV-induced wheezing illnesses in childhood, but not with those caused by RSV [67]. This is because chromosome 17q21 houses a group of genes, including ORMDL3 (orosomucoid-like 3), that play a role in modulating the risk of RV infection [67]. When ORMDL3 is suppressed, the expression of the RV receptor ICAM1 is significantly reduced, providing a clear explanation for the impact of chromosome 17q21 on RV susceptibility [68].

Another genetic predisposition associated with Cadherin-related family member 3 (CDHR3), a receptor for RV-C that mediates RV-C binding and replication in airway epithelia, appears to influence the risk of early RV-C infection and subsequent development of asthma. Changes in the CDHR3 gene that result in increased expression on cell surfaces and higher susceptibility to RV-C infections have been associated with an increased risk of chronic airway diseases [69,70,71]. On the other hand, variants that lead to lower cell surface density, such as the human specific Cys529 variant, seem to offer some protection against severe asthma episodes triggered by RV [72].

Finally, the season is an environmental factor that can impact the severity of symptoms. Specifically, RV infections are more likely to cause moderate to severe infections in the Winter, although the peak prevalence occurs in the Spring and Fall [73].

## 6. Long-Term Consequences of Rhinovirus Infection

Viral infections including those caused by RVs occurring during the neonatal period may trigger a Th2 immune response, with a negative impact on lung development, thereby exposing the patient to a significant risk of chronic respiratory disease, such as asthma [74]. The probability of having asthma is influenced by various factors, such as the intensity of the infection, the total number of infections and the stage of immune development, with exposure to a particular pathogen being a crucial factor [75]. Namely, the presence of RV in the respiratory secretions of infants and young children admitted to the hospital for wheezing, respiratory illness is connected to the development of asthma in the future [76], especially in those with a family history of asthma or allergies [53].

However, notwithstanding the growing body of research on the role of RV in aforementioned respiratory infections, the extent to which this virus contributes to the asthma development in otherwise healthy infants with no pre-existing risk factors is not yet fully elucidated. While some studies have indicated that RV-induced wheezing in early years of life may be a frank risk factor for subsequent occurrence of wheezing and asthma in both high-risk and low-risk populations of children [59,77], the mechanisms underlying this association remain elusive and unclear. Along those lines, De Winter et al. pinpointed RV-induced wheezing in early life as an inducer of subsequent wheezing and asthma [78], suggesting that this virus may play a role in the development of asthma even in infants with no family history of asthma, as well as no prior hospitalization for respiratory or wheezing illness.

Recently, several meta-analyses have been published that summarize the findings from the past thirty years of research, pointing to a connection between RV infection and the future development of asthma. First of those published by Liu et al. searched for studies published between 1988 and February 2017 and included 15 original articles which met the criteria [49]. This meta-analysis found that RV wheezing illness in the first 3 years of life was associated with an increased risk of wheezing/asthma in later life (with a relative risk of 2.00, 95% CI of 1.62–2.49 and *p* < 0.001) [49]. The most recent one that compared RV and RSV infected children showed that the RV-bronchiolitis group was more likely to develop recurrent wheeze (OR 4.11; 95% CI 2.24–7.56) and asthma (OR 2.72; 95% CI 1.48–4.99) than the RSV-bronchiolitis group [79].

Some authors suggest a difference in pathogenicity between groups of rhinoviruses, particularly with regards to the development of asthma in children. The data shows that children infected RV-C have a higher incidence of asthma compared to those infected with RV-A (42% vs. 23% and 55% vs. 36%, respectively) [80]. A recent study that compared infants infected with rhinovirus and those with RSV found that the risk of recurrent wheeze in infants with RV-A or RV-B infection was not significantly different (both log-rank *p* > 0.10); however, the risk was significantly higher in infants with RV-C bronchiolitis (log-rank *p* = 0.006), particularly in those with IgE sensitization (log-rank *p* = 0.01) [81]. Additionally, children who have bronchiolitis caused by RV-A and particularly RV-C tend to start using asthma control medication earlier and are more likely to still be using it four years later, in comparison to children with RSV-induced bronchiolitis [59].

## 7. Pathogenesis of Asthma and Recurrent Wheezing Caused by Rhinoviruses

Compared to other respiratory viruses, RV has minimal cytotoxic effects on airway epithelial cells. Its pathogenesis relies heavily on cytokines, and studies have identified correlations between respiratory symptoms and levels of cytokines such as interleukin (IL)-8, IL-4, IL-5, and IL-13 [82]. Numerous studies revealed two main characteristics in the immune response in asthmatics following infection with RV: an exaggerated activation of T2 immune pathways and a deficiency and/or delay in the antiviral immune response to RV infection [62]. In addition, according to a study by Rajput and colleagues, it has been demonstrated in an animal model that RV-C infection, which is more closely associated with the development of asthma, leads to an increase in Type 2 innate lymphoid cells and eosinophilic airway inflammation, in contrast to RV A [83]. This is in line with a broad range of serotypes (conferring only a limited immunologic cross-protection) and mutational flexibility that influences immune responses, as described in seminal papers regarding the discovery of discovery of RV-C [84,85]. Also, studies have identified how the asthma susceptibility gene product known as ‘human cadherin-related family member 3′ (or CDHR3) is responsible for mediating RV-C entry into host cells, suggesting that specific mutation could represent a risk factor for wheezing illnesses caused by this viral species [86].

Upon infection the respiratory epithelium by RV, release of the epithelial-derived cytokines IL-25 and IL-33 contribute to type 2 inflammation in individuals with asthma [82,87]. Specifically, Th2-cells and Innate Lymphoid Type-2 (ILC2) cells can be activated by these cytokines and TSLP (thymic stromal lymphopoietin) during infections. ILC2 cells then release IL-4, IL-5, IL-9, IL-13 and amphiregulin (Areg), and their overproduction has been observed following early-life respiratory viral infections [88]. The early over-expression of certain cytokines by ILC2 can result in eosinophilia (IL-5), increased mucus production (IL-13) and lung remodeling (Areg), which can lead to the development of asthma [88]. The clinical and biological characteristics of asthmatic children with RV infection are usually marked by the increase of polymorphonuclear leukocytes and Th2-related cytokines, especially with IL-5 and IL-13 [89]. Additionally, ILC2 has been found to induce adaptive Th2-Type immunity during acute exacerbation of chronic obstructive pulmonary disease (COPD) [90].

Moreover, the findings on animal model indicate that RV infection of the respiratory epithelium may also increase the risk of developing asthmatic lung inflammation by inhibiting tolerance to inhaled antigens through the simultaneous activation of thymic stromal lymphopoietin (TSLP), IL-33 and OX40L-TNF family ligand expressed on antigen-presenting cells [91].

There is a diminished and insufficient antiviral response to RV infection in atopic subjects [92]. It has been observed that atopic patients have reduced production of interferon (IFN-β and IFN-λ) compared to healthy subjects after RV infection [82]. More specifically, Balardo and co-authors have found that children with atopy and/or asthma had a significant decrease in interferon production induced by RV type 16, compared to non-atopic and non-asthmatic children [93]. This decreased antiviral response was accompanied with higher levels of viral RNA, epithelial damage, eosinophilia, IL-4 positivity and higher levels of total serum IgE [93].

Recent transcriptomic studies (bulk sequencing/single-cell sequencing) and proteomic studies have many implications for steering the future research in RV and children’s respiratory health. For example, by using a comparative transcriptomic analysis, Dissanayake et al. showed how genes related to interferon and chemokine production dominated the host response associated with RV [94]. Likewise, integrated-omics analysis is used to pinpoint biologically relevant RV bronchiolitis endotypes in infants at higher risk for recurrent wheeze and asthma development [95].

## 8. A Snapshot of Treatment Studies for Rhinovirus Infection

Direct-acting antivirals (DAAs), including capsid binders and inhibitors of viral enzymes, have been developed in recent decades to combat rhinovirus infections [96]. Capsid binders prevent viral uncoating and genome release by binding to a canyon in the external structure of the viral capsid protein VP1. Pleconaril is one of the first ones to exhibit good oral pharmacokinetic profile, as well as clinical efficacy in shortening the duration of the disease by about one day [97]. Unfortunately, moderate side effects related to the gastrointestinal system and the induction of contraceptive metabolism were also described [97]. A formula for intranasal application was also developed, which showed effectiveness in the prevention of rhinovirus infection [98] and further assessed in prevention of rhinovirus induced exacerbation of asthma (Study P04295, NCT00394914) (https://clinicaltrials.gov/ct2/show/NCT00394914, accessed on 21 March 2023). Perhaps the most important deficiency of capsid binders is that they do not inhibit the replication of RV-C [99], which is a significant trigger for the onset and recurrence of asthma.

Protease inhibitors such as rupintrivir (whose effectiveness in clinical research has not been verified) and polymerase inhibitors such as gemcitabine (an anticancer medication) are among the viral enzyme inhibitors [100,101]. Gemcitabine has shown promise as an antiviral drug, with a lower dose required for antiviral activity than for anticancer activity, suggesting a frank potential for an application that avoids the toxic effects of many anticancer drugs.

A different approach is related to host-targeting antivirals (HTAs). Physiological cholesterol-derived molecules, such as 25-hydroxycholesterol (25OHC) and 27-hydroxycholesterol (27OHC), recently emerged as broad-spectrum HTAs [102]. They show antiviral activities on both naked and enveloped viruses using different antiviral mechanisms [103,104,105,106]. More specifically, 25OHC acts as an HTA against rhinoviruses by inducing a delocalization of the oxysterol-binding protein to the Golgi vesicles, thereby leading to a reduction in phosphatidylinositol 4-phosphate (P14KB) on the endoplasmic reticulum membrane, which is fundamental for the recruitment of viral RNA-dependent RNA polymerase [107,108]. Additionally, P14kB inhibitors exhibit a wide-ranging antiviral impact [109]; nonetheless, their significant toxicity renders them unsuitable for clinical application at present [96,110].

To summarize, despite significant progress in the research of antiviral medications targeting RV, such as capsid binders which appear to hold the most potential, there is presently no dedicated treatment for RV.

## 9. Conclusions

Overall, the available evidence suggests that RV is a significant contributor to respiratory illness and may play a role in the development of asthma in both high-risk and low-risk populations of children. Hence, a paradigm shift entails moving from the view that RVs are only a common nuisance of childhood to a frank pathogen, especially in the presence of certain risk factors. Although this viral agent is still most frequently linked to the high burden of URTIs, the literature clearly shows its role in LRTIs such as pneumonia, bronchiolitis and several forms of severe respiratory disease. Moreover, there are notable long-term consequences of rhinovirus infection, primarily asthma and recurrent wheeze, with their distinct pathogenesis and pathophysiology. Further research is needed to fully understand the mechanisms underlying this association and to develop effective strategies for preventing and treating RV-induced respiratory illness and asthma.

## Figures and Tables

**Table 1:** Relevant meta-analyses exploring the prevalence of rhinoviruses in children with bronchiolitis and community-acquired pneumonia (CAP), as well as in patients with coronavirus disease 2019 (COVID-19).

Authors	Publication Year	Population Examined	Final Number of Included Studies	Sample Size	Rhinovirus Prevalence
Kenmoe et al.	2020	Children < 2 years with bronchiolitis	51	79,803	19.29% * (95% CI 16.67–22.04%) *
Pratt et al.	2022	Children with community-acquired pneumonia	186	152,209	22.1% (95% CI 19.5–24.7%)
Krubmein et al.	2023	Children and adults with COVID-19	59	149,319	1.32% (95% CI 1.15–1.52%)

* Denotes prevalence percentage that includes not only rhinovirus but other enteroviruses as well.

## Data Availability

Data sharing not applicable.

## References

[1] Gern J.E. (2010). The ABCs of rhinoviruses, wheezing, and asthma. J. Virol..

[2] Vandini S., Biaggi C., Fischer M., Lanari M. (2019). Impact of Rhinovirus Infections in Children. Viruses..

[3] Bizot E., Bousquet A., Charpié M., Coquelin F., Lefevre S., Le Lorier J., Patin M., Sée P., Sarfati E., Walle S. (2021). Rhinovirus: A Narrative Review on Its Genetic Characteristics, Pediatric Clinical Presentations, and Pathogenesis. Front. Pediatr..

[4] Neugebauer F., Bergs S., Liebert U.G., Hönemann M. (2022). Human Rhinoviruses in Pediatric Patients in a Tertiary Care Hospital in Germany: Molecular Epidemiology and Clinical Significance. Viruses..

[5] Price W.H. (1956). The isolation of a new virus associated with respiratory clinical disease in humans. Proc. Natl. Acad. Sci. USA.

[6] Gern J.E., Palmenberg A.C., Knipe D.M., Howley P.M. (2013). Rhinoviruses. Fields Virology.

[7] Jacobs S.E., Lamson D.M., George K.S., Walsh T.J. (2013). Human rhinoviruses. Clin. Microbiol. Rev..

[8] Simmonds P., Gorbalenya A.E., Harvala H., Hovi T., Knowles N.J., Lindberg A.M., Oberste M.S., Palmenberg A.C., Reuter G., Skern T. (2020). Recommendations for the nomenclature of enteroviruses and rhinoviruses. Arch. Virol..

[9] Royston L., Tapparel C. (2016). Rhinoviruses and respiratory enteroviruses: Not as simple as ABC. Viruses.

[10] Basnet S., Palmenberg A.C., Gern J.E. (2019). Rhinoviruses and Their Receptors. Chest.

[11] Palmenberg A.C., Gern J.E. (2015). Classification and evolution of human rhinoviruses. Methods Mol. Biol..

[12] Esneau C., Duff A.C., Bartlett N.W. (2022). Understanding Rhinovirus Circulation and Impact on Illness. Viruses.

[13] Lopez-Souza N., Favoreto S., Wong H., Ward T., Yagi S., Schnurr D., Finkbeiner W.E., Dolganov G.M., Widdicombe J.H., Boushey H.A. (2009). In vitro susceptibility to rhinovirus infection is greater for bronchial than for nasal airway epithelial cells in human subjects. J. Allergy Clin. Immunol..

[14] Giardina F.A.M., Piralla A., Ferrari G., Zavaglio F., Cassaniti I., Baldanti F. (2022). Molecular Epidemiology of Rhinovirus/Enterovirus and Their Role on Cause Severe and Prolonged Infection in Hospitalized Patients. Microorganisms.

[15] Savolainen-Kopra C., Korpela T., Simonen-Tikka M.L., Amiryousefi A., Ziegler T., Roivainen M., Hovi T. (2012). Single treatment with ethanol hand rub is ineffective against human rhinovirus hand washing with soap and water removes the virus efficiently. J. Med. Virol..

[16] Stott E.J., Heath G.F. (1970). Factors affecting the growth of rhinovirus 2 in suspension cultures of L132 cells. J. Gen. Virol..

[17] Papadopoulos N., Sanderson G., Hunter J., Johnston S. (1999). Rhinoviruses replicate effectively at lower airway temperatures. J. Med. Virol..

[18] Kreger J.E., Hershenson M.B. (2022). Effects of COVID-19 and Social Distancing on Rhinovirus Infections and Asthma Exacerbations. Viruses.

[19] Papadopoulos N.G., Bates P.J., Bardin P.G., Papi A., Leir S.H., Fraenkel D.J., Meyer J., Lackie P.M., Sanderson G., Holgate S.T. (2000). Rhinoviruses infect the lower airways. J. Infect. Dis..

[20] Mosser A.G., Vrtis R., Burchell L., Lee W.-M., Dick C.R., Weisshaar E., Bock D., Swenson C.A., Cornwell R.D., Meyer K.C. (2005). Quantitative and qualitative analysis of rhinovirus infection in bronchial tissues. Am. J. Respir. Crit. Care Med..

[21] Halabi K.C., Stockwell M.S., Alba L., Vargas C., Reed C., Saiman L., Mobile Surveillance for Acute Respiratory Infection/Influenza-like Illness in the Community (MoSAIC) Study Team (2022). Clinical and socioeconomic burden of rhinoviruses/enteroviruses in the community. Influenza Other Respir. Viruses..

[22] Jin X., Ren J., Li R., Gao Y., Zhang H., Li J., Zhang J., Wang X., Wang G. (2021). Global burden of upper respiratory infections in 204 countries and territories, from 1990 to 2019. EClinicalMedicine.

[23] Lim J.T., Tan K.B., Abisheganaden J., Dickens B.L. (2023). Forecasting upper respiratory tract infection burden using high-dimensional time series data and forecast combinations. PLoS Comput. Biol..

[24] Takashita E., Kawakami C., Momoki T., Saikusa M., Shimizu K., Ozawa H., Kumazaki M., Usuku S., Tanaka N., Okubo I. (2021). Increased risk of rhinovirus infection in children during the coronavirus disease-19 pandemic. Influenza Other Respir. Viruses.

[25] Nickbakhsh S., Mair C., Matthews L., Reeve R., Johnson P.C.D., Thorburn F., von Wissmann B., Reynolds A., McMenamin J., Gunson R.N. (2019). Virus-virus interactions impact the population dynamics of influenza and the common cold. Proc. Natl. Acad. Sci. USA.

[26] Dee K., Goldfarb D.M., Haney J., Amat J.A.R., Herder V., Stewart M., Szemiel A.M., Baguelin M., Murcia P.R. (2021). Human Rhinovirus Infection Blocks Severe Acute Respiratory Syndrome Coronavirus 2 Replication Within the Respiratory Epithelium: Implications for COVID-19 Epidemiology. J. Infect. Dis..

[27] Essaidi-Laziosi M., Alvarez C., Puhach O., Sattonnet-Roche P., Torriani G., Tapparel C., Kaiser L., Eckerle I. (2022). Sequential infections with rhinovirus and influenza modulate the replicative capacity of SARS-CoV-2 in the upper respiratory tract. Emerg. Microbes Infect..

[28] Kenmoe S., Kengne-Nde C., Ebogo-Belobo J.T., Mbaga D.S., Fatawou Modiyinji A., Njouom R. (2020). Systematic review and meta-analysis of the prevalence of common respiratory viruses in children < 2 years with bronchiolitis in the pre-COVID-19 pandemic era. PLoS ONE.

[29] Pratt M.T.G., Abdalla T., Richmond P.C., Moore H.C., Snelling T.L., Blyth C.C., Bhuiyan M.U. (2022). Prevalence of respiratory viruses in community-acquired pneumonia in children: A systematic review and meta-analysis. Lancet Child. Adolesc. Health.

[30] Krumbein H., Kümmel L.S., Fragkou P.C., Thölken C., Hünerbein B.L., Reiter R., Papathanasiou K.A., Renz H., Skevaki C. (2023). Respiratory viral co-infections in patients with COVID-19 and associated outcomes: A systematic review and meta-analysis. Rev. Med. Virol..

[31] Jartti T., Lehtinen P., Vuorinen T., Koskenvuo M., Ruuskanen O. (2004). Persistence of rhinovirus and enterovirus RNA after acute respiratory illness in children. J. Med. Virol..

[32] Heymann P.W., Platts-Mills T.A., Johnston S.L. (2005). Role of viral infections, atopy and antiviral immunity in the etiology of wheezing exacerbations among children and young adults. Pediatr. Infect. Dis. J..

[33] Singleton R.J., Bulkow L.R., Miernyk K., DeByle C., Pruitt L., Hummel K.B., Bruden D., Englund J.A., Anderson L.J., Lucher L. (2010). Viral respiratory infections in hospitalized and community control children in Alaska. J. Med. Virol..

[34] Calvo C., Casas I., Garcia-Garcia M.L., Pozo F., Reyes N., Cruz N., García-Cuenllas L., Pérez-Breña P. (2010). Role of rhinovirus C respiratory infections in sick and healthy children in Spain. Pediatr. Infect. Dis. J..

[35] Basharat U., Aiche M.M., Kim M.M., Sohal M., Chang E.H. (2019). Are rhinoviruses implicated in the pathogenesis of sinusitis and chronic rhinosinusitis exacerbations? A comprehensive review. Int. Forum Allergy Rhinol..

[36] Zhao Y., Shen J., Wu B., Liu G., Lu R., Tan W. (2018). Genotypic Diversity and Epidemiology of Human Rhinovirus Among Children With Severe Acute Respiratory Tract Infection in Shanghai, 2013–2015. Front. Microbiol..

[37] Ljubin-Sternak S., Meštrović T., Ivković-Jureković I., Kolarić B., Slović A., Forčić D., Tot T., Mijač M., Vraneš J. (2019). The Emerging Role of Rhinoviruses in Lower Respiratory Tract Infections in Children—Clinical and Molecular Epidemiological Study From Croatia, 2017-2019. Front. Microbiol..

[38] Esposito S., Daleno C., Prunotto G., Scala A., Tagliabue C., Borzani I., Fossali E., Pelucchi C., Principi N. (2013). Impact of viral infections in children with community-acquired pneumoniae: Results of a study of 17 respiratory viruses. Influenza Other Respir. Viruses.

[39] Ning G., Wang X., Wu D., Yin Z., Li Y., Wang H., Yang W. (2017). The etiology of community-acquired pneumonia among children under 5 years of age in mainland China, 2001–2015: A systematic review. Hum. Vaccines Immunother..

[40] Čivljak R., Tot T., Falsey A.R., Huljev E., Vraneš J., Ljubin-Sternak S. (2019). Viral pathogens associated with acute respiratory illness in hospitalized adults and elderly from Zagreb, Croatia, 2016 to 2018. J. Med. Virol..

[41] Esposito S., Daleno C., Tagliabue C., Scala A., Tenconi R., Borzani I., Fossali E., Pelucchi C., Piralla A., Principi N. (2012). Impact of rhinoviruses on pediatric community-acquired pneumonia. Eur. J. Clin. Microbiol. Infect. Dis..

[42] Hartiala M., Lahti E., Forsström V., Vuorinen T., Ruuskanen O., Peltola V. (2019). Characteristics of Hospitalized Rhinovirus-Associated Community-Acquired Pneumonia in Children, Finland, 2003-2014. Front. Med..

[43] Bahabri I., Abdulaal A., Alanazi T., Alenazy S., Alrumih Y., Alqahtani R., Bosaeed M., Al-Dorzi H.M. (2022). Characteristics, Management, and Outcomes of Community-Acquired Pneumonia Due to Human Rhinovirus-A Retrospective Study. Can. Respir. J..

[44] Tsagarakis N.J., Sideri A., Makridis P., Triantafyllou A., Stamoulakatou A., Papadogeorgaki E. (2018). Age-related prevalence of common upper respiratory pathogens, based on the application of the filmarray respiratory panel in a tertiary hospital in Greece. Medicine.

[45] Feddema J.J., Claassen E. (2020). Prevalence of Viral Respiratory Infections Amongst Asthmatics: Results of a Meta-Regression Analysis. Respir. Med..

[46] Kotaniemi-Syrjänen A., Reijonen T.M., Korhonen K., Waris M., Vainionpää R., Korppi M. (2008). Wheezing due to rhinovirus infection in infancy: Bronchial hyperresponsiveness at school age. Pediatr. Int..

[47] Turunen R., Koistinen A., Vuorinen T., Arku B., Söderlund-Venermo M., Ruuskanen O., Jartti T. (2014). The first wheezing episode: Respiratory virus etiology, atopic characteristics, and illness severity. Pediatr. Allergy Immunol..

[48] Jartti T., Gern J.E. (2017). Role of viral infections in the development and exacerbation of asthma in children. J. Allergy Clin. Immunol..

[49] Liu L., Pan Y., Zhu Y., Song Y., Su X., Yang L., Li M. (2017). Association between rhinovirus wheezing illness and the development of childhood asthma: A metaanalysis. BMJ Open.

[50] Drysdale S.B., Mejias A., Ramilo O. (2017). Rhinovirus—Not just the common cold. J. Infect..

[51] Jartti T., Lehtinen P., Vuorinen T., Ruuskanen O. (2009). Bronchiolitis: Age and previous wheezing episodes are linked to viral etiology and atopic characteristics. Pediatr. Infect. Dis. J..

[52] Hyvarinen M.K., Kotaniemi-Syrjanen A., Reijonen T.M., Korhonen K., Korppi M.O. (2005). Teenage asthma after severe early childhood wheezing: An 11-year prospective follow-up. Pediatr. Pulmonol..

[53] Jackson D.J., Gangnon R.E., Evans M.D., Roberg K.A., Anderson E.L., Pappas T.E., Printz M.C., Lee W.M., Shult P.A., Reisdorf E. (2008). Wheezing rhinovirus illnesses in early life predict asthma development in high-risk children. Am. J. Respir. Crit. Care Med..

[54] Midulla F., Nicolai A., Ferrara M., Gentile F., Pierangeli A., Bonci E., Scagnolari C., Moretti C., Antonelli G., Papoff P. (2014). Recurrent wheezing 36 months after bronchiolitis is associated with rhinovirus infections and blood eosinophilia. Acta Paediatr..

[55] Kusel M.M., de Klerk N.H., Holt P.G., Kebadze T., Johnston S.L., Sly P.D. (2006). Role of respiratory viruses in acute upper and lower respiratory tract illness in the first year of life: A birth cohort study. Pediatr. Infect. Dis. J..

[56] Jartti T., Smits H.H., Bønnelykke K., Bircan O., Elenius V., Konradsen J.R., Maggina P., Makrinioti H., Stokholm J., Hedlin G. (2019). EAACI task force on clinical practice recommendations on preschool wheeze. Bronchiolitis needs a revisit: Distinguishing between virus entities and their treatments. Allergy.

[57] Dumas O., Hasegawa K., Mansbach J.M., Sullivan A.F., Piedra P.A., Camargo C.A. (2019). Severe bronchiolitis profiles and risk of recurrent wheeze by age 3 years. J. Allergy Clin. Immunol..

[58] Takeyama A., Hashimoto K., Sato M., Sato T., Tomita Y., Maeda R., Ito M., Katayose M., Kawasaki Y., Hosoya M. (2014). Clinical and epidemiologic factors related to subsequent wheezing after virus-induced lower respiratory tract infections in hospitalized pediatric patients younger than 3 years. Eur. J. Pediatr..

[59] Bergroth E., Aakula M., Elenius V., Remes S., Piippo-Savolainen E., Korppi M., Piedra P.A., Bochkov Y.A., Gern J.E., Camargo C.A. (2020). Rhinovirus Type in Severe Bronchiolitis and the Development of Asthma. J. Allergy Clin. Immunol. Pract..

[60] Nanishi M., Chandran A., Li X., Stanford J.B., Alshawabkeh A.N., Aschner J.L., Dabelea D., Dunlop A.L., Elliott A.J., Gern J.E. (2022). Association of Severe Bronchiolitis during Infancy with Childhood Asthma Development: An Analysis of the ECHO Consortium. Biomedicines.

[61] van Benten I., Koopman L., Niesters B., Hop W., van Middelkoop B., de Waal L., van Drunen K., Osterhaus A., Neijens H., Fokkens W. (2003). Predominance of rhinovirus in the nose of symptomatic and asymptomatic infants. Pediatr. Allergy Immunol..

[62] Jackson D.J., Gern J.E. (2022). Rhinovirus infections and their roles in asthma: Etiology and exacerbations. J. Allergy Clin. Immunol. Pract..

[63] Nakagome K., Bochkov Y.A., Ashraf S., Brockman-Schneider R.A., Evans M.D., Pasic T.R., Gern J.E. (2014). Effects of rhinovirus species on viral replication and cytokine production. J. Allergy Clin. Immunol..

[64] Choi T., Devries M., Bacharier L.B., Busse W., Camargo C.A., Cohen R., Demuri G.P., Evans M.D., Fitzpatrick A.M., Gergen P.J. (2021). Enhanced Neutralizing Antibody Responses to Rhinovirus C and Age-Dependent Patterns of Infection. Am. J. Respir. Crit. Care Med..

[65] Bruning A.H.L., Thomas X.V., van der Linden L., Wildenbeest J.G., Minnaar R.P., Jansen R.R., de Jong M.D., Sterk P.J., van der Schee M.P., Wolthers K.C. (2015). Clinical, virological and epidemiological characteristics of rhinovirus infections in early childhood: A comparison between non-hospitalised and hospitalised children. J. Clin. Virol..

[66] Sayama A., Okamoto M., Tamaki R., Saito-Obata M., Saito M., Kamigaki T., Sayama Y., Lirio I., Manalo J.I.G., Tallo V.L. (2022). Comparison of Rhinovirus A-, B-, and C-Associated Respiratory Tract Illness Severity Based on the 5′-Untranslated Region Among Children Younger Than 5 Years. Open Forum Infect. Dis..

[67] Çalışkan M., Bochkov Y.A., Kreiner-Møller E., Bønnelykke K., Stein M.M., Du G., Bisgaard H., Jackson D.J., Gern J.E., Lemanske R.F. (2013). Rhinovirus wheezing illness and genetic risk of childhood-onset asthma. N. Engl. J. Med..

[68] Zhang Y., Willis-Owen S.A.G., Spiegel S., Lloyd C.M., Moffatt M.F., Cookson W.O.C.M. (2019). The ORMDL3 Asthma Gene Regulates ICAM1 and Has Multiple Effects on Cellular Inflammation. Am. J. Respir. Crit. Care Med..

[69] Bønnelykke K., Sleiman P., Nielsen K., Kreiner-Møller E., Mercader J.M., Belgrave D. (2014). A genome-wide association study identifies CDHR3 as a susceptibility locus for early childhood asthma with severe exacerbations. Nat. Genet..

[70] Kanazawa J., Masuko H., Yatagai Y., Sakamoto T., Yamada H., Kaneko Y. (2017). Genetic association of the functional CDHR3 genotype with early-onset adult asthma in Japanese populations. Allergol. Int..

[71] Shigemasa R., Masuko H., Hyodo K., Kitazawa H., Kanazawa J., Yatagai Y., Iijima H., Naito T., Saito T., Hirota T. (2020). Genetic impact of CDHR3 on the adult onset of asthma and COPD. Clin. Exp. Allergy.

[72] Palmenberg A.C. (2017). Rhinovirus C, Asthma, and Cell Surface Expression of Virus Receptor CDHR3. J. Virol..

[73] Lee W.M., Lemanske R.F., Evans M.D., Vang F., Pappas T., Gangnon R., Jackson D.J., Gern J.E. (2012). Human rhinovirus species and season of infection determine illness severity. Am. J. Respir. Crit. Care Med..

[74] Restori K.H., Srinivasa B.T., Ward B.J., Fixman E.D. (2018). Neonatal Immunity, Respiratory Virus Infections, and the Development of Asthma. Front. Immunol..

[75] Gern J.E. (2004). Viral respiratory infection and the link to asthma. Pediatr. Infect. Dis. J..

[76] Kotaniemi-Syrjanen A., Vainionpaa R., Reijonen T.M., Waris M., Korhonen K., Korppi M. (2003). Rhinovirus-induced wheezing in infancy—The first sign of childhood asthma?. J. Allergy Clin. Immunol..

[77] Da Silva Sena C.R., Morten M., Meredith J., Kepreotes E., Murphy V.E., Gibson G.P., Robinson P.D., Sly P.D., Whitehead B., Karmaus W. (2021). Rhinovirus bronchiolitis, maternal asthma, and the development of asthma and lung function impairments. Pediatr. Pulmonol..

[78] De Winter J.J., Bont L., Wilbrink B., van der Ent C.K., Smit H.A., Houben M.L. (2015). Rhinovirus wheezing illness in infancy is associated with medically attended third year wheezing in low risk infants: Results of a healthy birth cohort study. Immun. Inflamm. Dis..

[79] Makrinioti H., Hasegawa K., Lakoumentas J., Xepapadaki P., Tsolia M., Castro-Rodriguez J.A., Feleszko W., Jartti T., Johnston S.L., Bush A. (2022). The role of respiratory syncytial virus- and rhinovirus-induced bronchiolitis in recurrent wheeze and asthma-A systematic review and meta-analysis. Pediatr. Allergy Immunol..

[80] Miller E.K., Edwards K.M., Weinberg G.A., Iwane M.K., Griffin M.R., Hall C.B., Zhu Y., Szilagyi P.G., Morin L.L., Heil L.H. (2009). New Vaccine Surveillance Network. A novel group of rhinoviruses is associated with asthma hospitalizations. J. Allergy Clin. Immunol..

[81] Hasegawa K., Mansbach J.M., Bochkov Y.A., Gern J.E., Piedra P.A., Bauer C.S., Teach S.J., Wu S., Sullivan A.F., Camargo C.A. (2019). Association of Rhinovirus C Bronchiolitis and Immunoglobulin E Sensitization During Infancy with Development of Recurrent Wheeze. JAMA Pediatr..

[82] Liew K.Y., Koh S.K., Hooi S.L., Ng M.K.L., Chee H.Y., Harith H.H., Israf D.A., Tham C.L. (2022). Rhinovirus-Induced Cytokine Alterations with Potential Implications in Asthma Exacerbations: A Systematic Review and Meta-Analysis. Front. Immunol..

[83] Rajput C., Han M., Ishikawa T., Lei J., Goldsmith A.M., Jazaeri S., Stroupe C.C., Bentley J.K., Hershenson M.B. (2021). Rhinovirus C Infection Induces Type 2 Innate Lymphoid Cell Expansion and Eosinophilic Airway Inflammation. Front. Immunol..

[84] Arden K.E., McErlean P., Nissen M.D., Sloots T.P., Mackay I.M. (2006). Frequent detection of human rhinoviruses, paramyxoviruses, coronaviruses, and bocavirus during acute respiratory tract infections. J. Med. Virol..

[85] Lau S.K., Yip C.C., Woo P.C., Yuen K.Y. (2010). Human rhinovirus C: A newly discovered human rhinovirus species. Emerg. Health Threat. J..

[86] Bochkov Y.A., Watters K., Ashraf S., Griggs T.F., Devries M.K., Jackson D.J., Palmenberg A.C., Gern J.E. (2015). Cadherin-related family member 3, a childhood asthma susceptibility gene product, mediates rhinovirus C binding and replication. Proc. Natl. Acad. Sci. USA.

[87] Beale J., Jayaraman A., Jackson D.J., Macintyre J.D., Edwards M.R., Walton R.P., Zhu J., Ching Y.M., Shamji B., Edwards M. (2014). Rhinovirus-induced IL-25 in asthma exacerbation drives type 2 immunity and allergic pulmonary inflammation. Sci. Transl. Med..

[88] Fonseca W., Lukacs N.W., Elesela S., Malinczak C.A. (2021). Role of ILC2 in Viral-Induced Lung Pathogenesis. Front. Immunol..

[89] Nguyen-Thi-Dieu T., Le-Thi-Thu H., Le-Thi-Minh H., Pham-Nhat A., Duong-Quy S. (2018). Study of Clinical Characteristics and Cytokine Profiles of Asthmatic Children with Rhinovirus Infection during Acute Asthma Exacerbation at National Hospital of Pediatrics. Can. Respir. J..

[90] Jiang M., Liu H., Li Z., Wang J., Zhang F., Cao K., Li F., Ding J. (2019). ILC2s Induce Adaptive Th2-Type Immunity in Acute Exacerbation of Chronic Obstructive Pulmonary Disease. Mediat. Inflamm..

[91] Mehta A.K., Duan W., Doerner A.M., Traves S.L., Broide D.H., Proud D., Zuraw B.L., Croft M. (2016). Rhinovirus infection interferes with induction of tolerance to aeroantigens through OX40 ligand, thymic stromal lymphopoietin, and IL-33. J. Allergy Clin. Immunol..

[92] Xatzipsalti M., Psarros F., Konstantinou G., Gaga M., Gourgiotis D., Saxoni-Papageorgiou P., Papadopoulos N.G. (2008). Modulation of the epithelial inflammatory response to rhinovirus in an atopic environment. Clin. Exp. Allergy.

[93] Baraldo S., Contoli M., Bonato M., Snijders D., Biondini D., Bazzan E., Cosio M.G., Barbato A., Papi A., Saetta M. (2018). Deficient Immune Response to Viral Infections in Children Predicts Later Asthma Persistence. Am. J. Respir. Crit. Care Med..

[94] Dissanayake T.K., Schäuble S., Mirhakkak M.H., Wu W.L., Ng A.C., Yip C.C.Y., López A.G., Wolf T., Yeung M.L., Chan K.H. (2020). Comparative Transcriptomic Analysis of Rhinovirus and Influenza Virus Infection. Front. Microbiol..

[95] Raita Y., Camargo C.A., Bochkov Y.A., Celedón J.C., Gern J.E., Mansbach J.M., Rhee E.P., Freishtat R.J., Hasegawa K. (2021). Integrated-omics endotyping of infants with rhinovirus bronchiolitis and risk of childhood asthma. J. Allergy Clin. Immunol..

[96] Bauer L., Lyoo H., van der Schaar H.M., Strating J.R., van Kuppeveld F.J. (2017). Direct-acting antivirals and host-targeting strategies to combat enterovirus infections. Curr. Opin. Virol..

[97] Pevear D.C., Hayden F.G., Demenczuk T.M., Barone L.R., McKinlay M.A., Collett M.S. (2005). Relationship of pleconaril susceptibility and clinical outcomes in treatment of common colds caused by rhinoviruses. Antimicrob. Agents Chemother..

[98] Hayden F.G., Hipskind G.J., Woerner D.H., Eisen G.F., Janssens M., Janssen P.A., Andries K. (1995). Intranasal pirodavir (R77,975) treatment of rhinovirus colds. Antimicrob. Agents Chemother..

[99] Mello C., Aguayo E., Rodriguez M., Lee G., Jordan R., Cihlar T., Birkus G. (2014). Multiple classes of antiviral agents exhibit in vitro activity against human rhinovirus type C. Antimicrob. Agents Chemother..

[100] Binford S.L., Maldonado F., Brothers M.A., Weady P.T., Zalman L.S., Meador J.W., Matthews D.A., Patick A.K. (2005). Conservation of amino acids in human rhinovirus 3C protease correlates with broad-spectrum antiviral activity of rupintrivir, a novel human rhinovirus 3C protease inhibitor. Antimicrob. Agents Chemother..

[101] Zhang Z., Yang E., Hu C., Cheng H., Chen C.Y., Huang D., Wang R., Zhao Y., Rong L., Vignuzzi M. (2017). Cell-Based High-Throughput Screening Assay Identifies 2′,2′-Difluoro-2′-deoxycytidine Gemcitabine as a Potential Antipoliovirus Agent. ACS Infect. Dis..

[102] Lembo D., Cagno V., Civra A., Poli G. (2016). Oxysterols: An emerging class of broad spectrum antiviral effectors. Mol. Asp. Med..

[103] Civra A., Cagno V., Donalisio M., Biasi F., Leonarduzzi G., Poli G., Lembo D. (2014). Inhibition of pathogenic non-enveloped viruses by 25-hydroxycholesterol and 27-hydroxycholesterol. Sci. Rep..

[104] Civra A., Francese R., Gamba P., Testa G., Cagno V., Poli G., Lembo D. (2018). 25-Hydroxycholesterol and 27-hydroxycholesterol inhibit human rotavirus infection by sequestering viral particles into late endosomes. Redox Biol..

[105] Gomes B., Sanna G., Madeddu S., Hollmann A., Santos N.C. (2019). Combining 25-Hydroxycholesterol with an HIV Fusion Inhibitor Peptide: Interaction with Biomembrane Model Systems and Human Blood Cells. ACS Infect. Dis..

[106] Zu S., Deng Y.Q., Zhou C., Li J., Li L., Chen Q., Li X.F., Zhao H., Gold S., He J. (2020). 25-Hydroxycholesterol is a potent SARS-CoV-2 inhibitor. Cell Res..

[107] Roulin P.S., Lötzerich M., Torta F., Tanner L.B., van Kuppeveld F.J., Wenk M.R., Greber U.F. (2014). Rhinovirus uses a phosphatidylinositol 4-phosphate/cholesterol counter-current for the formation of replication compartments at the ER-Golgi interface. Cell Host Microbe.

[108] Civra A., Costantino M., Cavalli R., Adami M., Volante M., Poli G., Lembo D. (2022). 27-Hydroxycholesterol inhibits rhinovirus replication in vitro and on human nasal and bronchial histocultures without selecting viral resistant variants. Antivir. Res..

[109] Rutaganira F.U., Fowler M.L., McPhail J.A., Gelman M.A., Nguyen K., Xiong A., Dornan G.L., Tavshanjian B., Glenn J.S., Shokat K.M. (2016). Design and Structural Characterization of Potent and Selective Inhibitors of Phosphatidylinositol 4 Kinase IIIβ. J. Med. Chem..

[110] Lamarche M.J., Borawski J., Bose A., Capacci-Daniel C., Colvin R., Dennehy M., Ding J., Dobler M., Drumm J., Gaither L.A. (2012). Anti-hepatitis C virus activity and toxicity of type III phosphatidylinositol-4-kinase beta inhibitors. Antimicrob. Agents Chemother..

